# The Role of Subjective Experiences in Conflict Tasks: A Review

**DOI:** 10.5334/pb.508

**Published:** 2021-02-08

**Authors:** Laurence Questienne, Jean-Philippe van Dijck, Wim Gevers

**Affiliations:** 1 Center for Research in Cognition & Neurosciences (CRCN) and UNI. Université Libre de Bruxelles, Brussels, Belgium; 2 Aix-Marseille Université, CNRS, LNC, UMR 7291, Laboratoire de Neurosciences Cognitives, Marseille, France; 3 Department of Applied Psychology, Thomas More University of Applied Sciences, Antwerp, Belgium; 4 Department of Experimental Psychology, Ghent University, Ghent, Belgium

**Keywords:** subjective experiences, metacognition, cognitive control

## Abstract

Cognitive control research is concerned with the question how we install adaptive behaviour in the case of (cognitive) conflict. In this review we focus on the role that awareness of this conflict plays in our ability to exert cognitive control. We will argue that visual conflict is not the only building block of metacognitive experiences of conflict and discuss how they are related to cognitive control. So, a first aim of the current review is to understand how these different metacognitive judgements are created. To do so, we draw some remarkable parallels with research on metacognition in decision making and memory research. Next, we elaborate on the relationship between metacognition and adaptive behaviour, with a specific focus on the role of subjective experiences in the Gratton effect. The grey areas that persist in the current literature are highlighted. In addition to deciphering the mechanisms of metacognitive judgements in cognitive control, this overview also aims to further enlarge our understanding of metacognitive abilities at a more general level.

Imagine waiting at a red traffic light where a policeman is facing you holding one hand up. When the traffic light turns green, you may automatically tighten the muscles of your right leg despite the policeman is still holding his hand up. When you become aware of this, you will probably pay increased attention to the instructions of the policeman to avoid accidents. There are a lot of everyday situations where our subjective experiences seem powerful at signaling the need to control and adapt our strategies to achieve our goal. The ultimate aim of this review is to understand which mechanisms underlie the emergence of such subjective experiences and to indicate their potential role in cognitive control and behavioral adaptation. In this first part, we describe the most important theories on cognitive control and explore whether awareness of conflicting information in the stimulus (e.g. are you aware that policeman and traffic lights indicate different actions) is needed to install adaptation. We will argue that, even if participants are not aware of the conflicting information, it is still possible that they have a subjective experience of difficulty, related to this unconscious conflict (e.g. you feel that you want to break and start riding at the same time). The second part of this review will focus on the question how such metacognitive judgements of difficulty are created. To do so, insights from studies on metacognition in memory and decision-making literature will be used. In the last part we consider the possible function of such metacognitive judgements for cognitive adaptation.

## Cognitive control and Conflict Tasks: the case of the Gratton effect

Cognitive control refers to the ability to adjust our information processing and our actions according to our goals in a complex environment that contains conflicting information (Miller & Cohen, 2001). Cognitive control is especially important to overrule automatic or usual responses that are inadequate in a given context (Botvinick, Braver, Barch, Carter, & Cohen, 2001; Miller & Cohen, 2001). Take another look at the driving example above. Most of the time, responding to the color of the traffic light is the obvious strategy to follow to start driving. However, this is no longer true in the presence of a policeman. In this case, you need to ignore the traffic light and to follow the instructions of the policeman. The processing of the information available in the environment needs to be adapted based on the context to modify behavior in agreement with our current goals.

Cognitive control implies different cognitive functions (e.g., cognitive flexibility, response inhibition, orienting of attention, etc.; see e.g., Diamond, 2013) studied through different experimental paradigms. For instance, in task-switching paradigms, participants are asked to switch from one task to another depending on the context (for an overview, see Kiesel et al., 2010). In stop-signal paradigms, participants inhibit an already prepared motor response when a stop-signal occurs (e.g., Logan, 1982). In the current work, we are especially interested in paradigms that ask participants to deal with conflicting responses generated by automatically processed but in that specific context, irrelevant information. We group these paradigms under the generic term of “conflict tasks”. Conflict tasks use stimuli with task- relevant and task-irrelevant dimensions. Typically, the task irrelevant dimension contains highly familiar information which is processed automatically. As such, when participants respond to the task-relevant dimension, they inhibit the task-irrelevant dimension. A prototypical example of such a conflict task is the Stroop task (Stroop, 1935). In this task, participants are presented with colour words presented in a certain colour (e.g. the word ‘green’ presented in the colour ‘red’). Participants must name the colour of the ink (red) while ignoring the colour word (green). Colour and colour words can be congruent (e.g. *blue* written in blue) or incongruent (e.g. *blue* written in red). Because (in literate people), reading is an automatic process, participants are distracted by the meaning of the incongruent word. As a result, participants make more errors and are slower to name the colour ink on incongruent compared to congruent trials (e.g., MacLeod, 1991).

**Figure 1:**
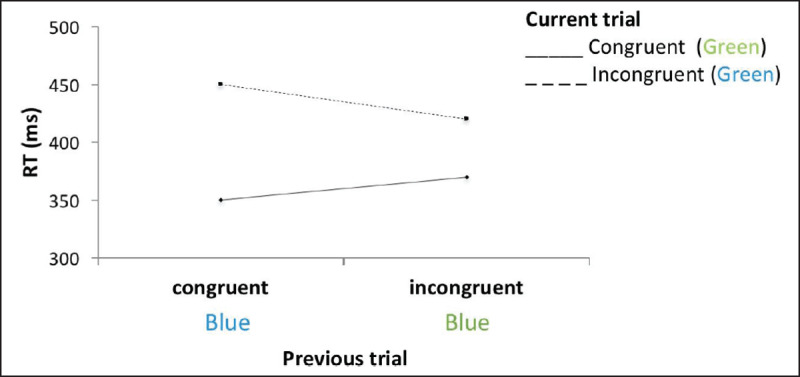
Classical Gratton effect. After an incongruent trial, the difference between congruent and incongruent trials is reduced (simulated data for illustrative purposes).

It has consistently been shown that the congruency effect is smaller after incongruent compared to after congruent trials (Gratton et al., 1992; see ***Figure 1***). This sequential modulation is known as the “Gratton effect”, also called the “congruency sequence effect” or “conflict adaptation”. It is often interpreted as the result of a modulation of information processing and response selection (e.g., Egner & Hirsch, 2005; Nigbur, Schneider, Sommer, Dimigen, & Stürmer, 2015; Stürmer et al., 2002) in response to task demands. In other words, the Gratton effect would be a hallmark of top-down cognitive control meaning that more control is engaged after conflict is detected by the conflict monitoring unit of the participant (Botvinick, Braver, Barch, Carter, & Cohen, 2001). This effect is not only present in the Stroop task (e.g., Kerns et al., 2004), but observed across different types of conflict tasks: e.g. the Flanker task (e.g., Gratton et al., 1992), the Simon task (e.g., Notebaert & Verguts, 2011a; Stürmer, Leuthold, Soetens, Schröter, & Sommer, 2002) and in experiments using priming (e.g., Kunde, 2003; van Gaal, Lamme, & Ridderinkhof, 2010).

In its original description, Gratton (1992) suggested that the effect results from an adaptation based on stimulus expectancies. People would expect congruency repetitions rather than congruency alternations. Consequently, after an incongruent trial, participants would focus more on the relevant dimension because they expect another incongruent trial. However, empirical studies show that the influence of such expectancies remains limited to situations that strongly induce or explicitly cue these expectancies (e.g., Duthoo, Abrahamse, Braem, & Notebaert, 2013; Duthoo & Notebaert, 2012; Duthoo, Wühr, & Notebaert, 2013; Jiménez & Méndez, 2013). In other words, repetition expectancy is not sufficient to explain all the Gratton effects reported in the literature (for an overview, see also Duthoo, Abrahamse, Braem, Boehler, & Notebaert, 2014).

**Figure 2:**
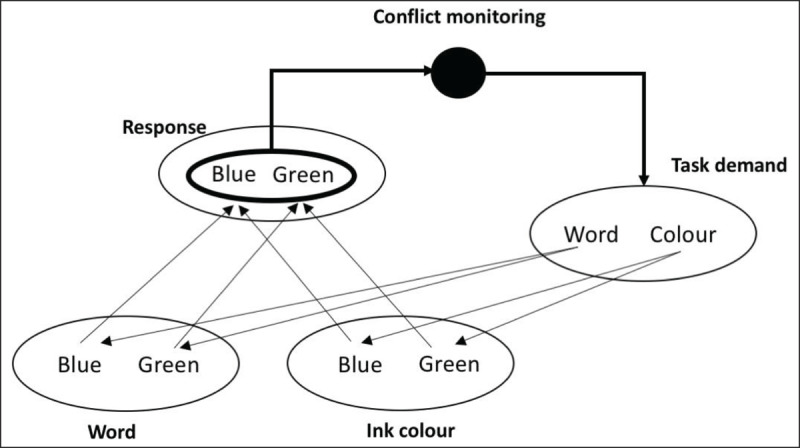
Schematic representation of the conflict monitoring theory. In a Stroop task, the ink colour and the word activate their corresponding response. In case of incongruent trials, competition is detected between the responses activated by the task-relevant and by the task- irrelevant dimension. As a result, attention on the task-relevant dimension is increased on the next trial. (adapted from Botvinick, Braver, Barch, Carter & Cohen, 2001).

The conflict monitoring theory (Botvinick et al., 2001; see also Botvinick, 2007; Botvinick, Cohen, & Carter, 2004; see ***Figure 2***) provides with a theory on when information processing is biased towards the processing of the relevant information and the suppression of the irrelevant information. On incongruent trials, competitive responses are activated by the task-relevant and the task-irrelevant stimulus dimension. According to the conflict monitoring theory, a monitoring system detects this co-activation of competitive responses, which is called response conflict. In this theory it is assumed that at the cerebral level, monitoring is done by the posterior medial frontal cortex (pMFC) and, more precisely, by the Anterior Cingulate Cortex (ACC). When a high level of response conflict is detected, the ACC sends a signal to the dorso-lateral prefrontal cortex (DLPFC) that induces cognitive control. This cognitive control biases information processing in favour of the relevant dimension on the next trial. This model received empirical support from electrophysiological and neuroimaging studies (Kerns, 2006; Kerns et al., 2004; Yeung, Botvinick, & Cohen, 2004), but it also demonstrates some weaknesses. For instance, how exactly the bias in information processing is implemented, must still be deciphered (e.g., Abrahamse, Braem, Notebaert, & Verguts, 2016). Also, whether the ACC really detects response conflict or other aspects (like time on task or error likelihood) remains highly debated in the literature (e.g., Alexander & Brown, 2011; Botvinick, 2007; Burle, Allain, Vidal, & Hasbroucq, 2005; Silvetti, Seurinck, & Verguts, 2011). In any case, the conflict monitoring theory highlights an important component of cognitive control: cognitive control implies continuous monitoring of the ongoing action to detect situations that require adaptation. This hypothesis that a system continuously monitors our actions is shared with other theories of adaptive behaviour, (for an overview, see Ullsperger, Danielmeier, & Jocham, 2014).

Other theories argue that the Gratton effect is triggered by bottom-up factors instead of top-down control (for an overview, see also Duthoo et al., 2014). In this context, the feature integration account suggests that the Gratton effect is the result from low-level stimulus repetition effects (e.g., Hommel, Proctor, & Vu, 2004; Mayr, Awh, & Laurey, 2003). According to this account, on every trial, stimulus and response features are temporarily associated. If, on the next trial, the association is maintained, processing will be relatively easy. If the association is violated, responses will slow down, and more errors will be made. A Gratton effect is observed because the associations are maintained better with congruency repetitions than with congruency alternations. Another account, the contingency learning account (Schmidt, 2013; Schmidt & Besner, 2008; Schmidt, Crump, Cheesman, & Besner, 2007; Schmidt & De Houwer, 2011), directly follows from these attempts to control for such bottom-up sequential effects. In most of the studies using four-choice congruency tasks, to maintain 50% of congruent trials, an irrelevant dimension (e.g., the word *blue*) is more often associated with a congruent response (e.g., *blue* written in blue) than with an incongruent response (e.g., *blue* written in red) (Schmidt, 2013). Participants learn such contingencies and it was observed that contingency learning boosts the Gratton effect (Schmidt et al., 2007).

However, even when both stimulus repetitions and contingency are controlled for, a Gratton effect is still observed (e.g., Kim & Cho, 2014; Notebaert, Gevers, Verbruggen, & Liefooghe, 2006; Schmidt & Weissman, 2014). Currently the consensus seems to be that both bottom-up (associative) and top- down (conflict adaptation) factors contribute to the Gratton effect (Duthoo, Abrahamse, Braem, Boehler, & Notebeaert, 2014) and that their effects can be disentangled (Braem et al., 2019; Egner, 2008).

## The awareness of conflict inducing stimuli and cognitive control

The definition of consciousness is by-itself a matter of debate (e.g., Baars & Laureys, 2005; Block, 2005; Kouider, de Gardelle, Sackur, & Dupoux, 2010). For the current purpose, we rely on a definition given by Lau and Rosenthal (2011) who use the term “consciousness” (also “conscious awareness”) to refer to mental states that occur with a subjective experience. A subjective experience is “what it feels like” to be in a certain state (Nagel, 1974; see also Glossary, Table I-1, p. 24). In line with Lau and Rosenthal (2011), we assume that conscious mental states can be explicitly reported. However, it is possible that this explicit report is limited merely because of a lack of words (e.g., Kouider et al., 2010). For instance, a consciously experienced smell can be difficult to verbalise because there is no verbal “label” corresponding with the subjective experience that it generates.

The nature of the relationship between consciousness and cognitive control is highly debated in the literature (e.g., Hommel, 2007, 2013, 2017; Kunde, Reuss, & Kiesel, 2012; Mayr, 2004; van Gaal, De Lange, & Cohen, 2012). One major question is whether awareness of the events that call for cognitive control is necessary to trigger cognitive and behavioural adaptation. One of the most influential theories of consciousness, the Global Neuronal Network theory (Dehaene & Naccache, 2001), makes a direct theoretical link between consciousness and cognitive control. This theory suggests that different cerebral networks can unconsciously process information. Routine actions, such as subliminal visual processing (e.g., Vorberg, Mattler, Heinecke, Schmidt, & Schwarsbach, 2003), can be performed by such modular networks. Importantly, information becomes conscious when its processing is amplified by attention, making it available in a global workspace distributed across the entire brain. Once in the global workspace, the information becomes available for verbal report and for novel or unusual processing that is mandatory for cognitive control. This model therefore takes a strong position. Events can trigger cognitive control only when they are represented in the Global Neuronal Workspace. In other words, cognitive control on an event can only be excerted if we are conscioulsly aware of the event. Supporting this relation, some results suggested that the ACC, the brain structure considered the heart of the monitoring process in the conflict monitoring theory, would be closely related to conscious experience (e.g., Dehaene et al., 2003; Dehaene & Naccache, 2001; Mulert, Menzinger, Leicht, Pogarell, & Hegerl, 2005).

However, this latter idea on the relationship between conscious awareness and cognitive control is far from gaining unanimous support. Firstly, all theories on consciousness do not necessarily assume such a strong relationship between consciousness and cognitive control (see Lau & Rosenthal, 2011, p. 366 for a comparison between several theories of consciousness). For instance, other influential views of consciousness are the Higher-Order theories of consciousness (for overviews, see Lau & Rosenthal, 2011; Rosenthal, 2004). These theories share the idea that consciousness implies a higher-order representation of a first-order state. Being aware of a visual stimulus implies having a higher-order representation of one’s self seeing this visual stimulus. However, these theories do not make explicit predictions about the relationship between consciousness of conflict and cognitive control. Second, the relations that have been observed between consciousness and cognitive control are correlational rather than causal (Hommel, 2013, 2017). Third, several empirical studies (for overviews, see Kunde et al., 2012; van Gaal et al., 2012) suggested that cognitive control can occur while being unaware of the stimulus that triggered this control (e.g., Hughes, Velmans, & De Fockert, 2009; Mattler, 2003, 2006; van Gaal, Lamme, Fahrenfort, & Ridderinkhof, 2011; van Gaal, Ridderinkhof, Fahrenfort, Scholte, & Lamme, 2008; van Gaal, Ridderinkhof, van den Wildenberg, & Lamme, 2009). For instance, Mattler (2003, 2006) observed that unconscious cues can facilitate switching form one task to another and van Gaal et al. (2009) observed that inhibition of motor responses can be triggered by an unconscious stimulus. These points challenge the hypothesis of a strong functional relationship between consciousness and cognitive control.

**Figure 3:**
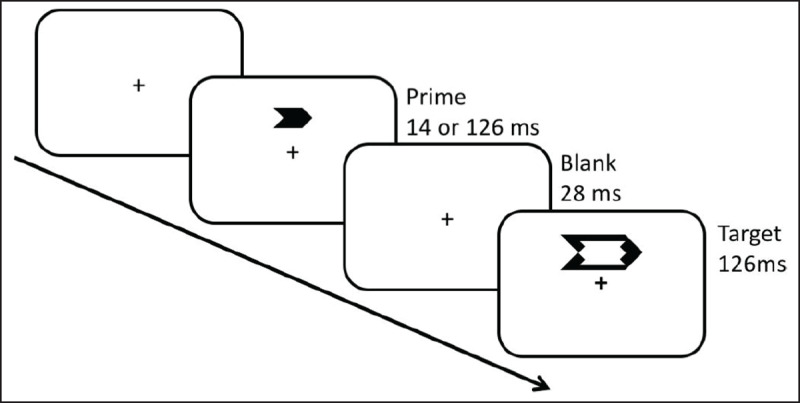
Arrow priming conflict task. Example of congruent trial in a subliminal priming conflict task. A prime arrow is presented subliminally or supraliminal before the target arrow (adapted from Kunde, 2003).

In the specific case of the Gratton effect, researchers tried to determine whether awareness of stimulus congruency was necessary for the effect to occur (for an overview, see Desender & Van den Bussche, 2012). To respond to this question, subliminal priming conflict tasks were used (Ansorge, Fuchs, Shah, & Kunde, 2011; Desender, Van Lierde, Van den Bussche, Reynvoet, & Sommer, 2013; Francken, van Gaal, & de Lange, 2011; Frings & Wentura, 2008; Greenwald, Draine, & Abrams, 1996; Jiang, Zhang, & van Gaal, 2015; Kunde, 2003; van Gaal et al., 2010). For instance, Kunde (2003) used an arrow priming conflict task (see ***Figure 3***) in which participants responded to the direction of a target arrow that could point to the left or to the right (see also Vorberg et al., 2003). Importantly, before the target, a prime arrow appeared that could point in the same or in the opposite direction of the target, generating a congruency effect. The crucial point was that in half of the trials, primes were presented so fast that they were not consciously perceived by the participants (i.e., subliminal prime) (Kunde, 2003, Experiment 2). In the other half of the trials, the primes were consciously detectable (i.e., supraliminal prime). Congruency effects were observed with both subliminal and supraliminal primes. Similarly, the Gratton effect was independent of the conscious awareness of the prime on the *current* trial. However, crucially, the conscious awareness of the prime on the *previous* trial mattered. The Gratton effect was observed only if the prime on the *previous* trial was consciously perceived. Similar results were obtained with other types of subliminal stimuli (e.g., Ansorge et al., 2011; Frings & Wentura, 2008; Greenwald et al., 1996). This led to the conclusion that conscious awareness of stimulus congruency is necessary to trigger cognitive control mechanisms responsible for the Gratton effect.

Contradictory to these first studies, other researchers (e.g., Desender et al., 2013; Jiang et al., 2015; van Gaal et al., 2010) also observed a Gratton effect after subliminal primes in a very similar priming task as the one used by Kunde (2003). The main difference was that the inter-stimulus interval was reduced and the warning signal indicating the beginning of the trial was removed. According to Van Gaal et al. (2010), the use of a long inter-stimulus interval and a warning signal could have made the participants release their attention during the inter-stimulus interval. As traces of subliminal primes are weak, releasing attention during the inter-stimulus interval would make the traces to disappear before the next trial.

Thus, it seems that conscious awareness of stimulus congruency is not necessary for a Gratton effect. An alternative explanation remains possible, however. Even if participants are not consciously aware of the stimulus congruency, they could still have a subjective experience related to some effects or by-products of this congruency. For instance, participants could consciously feel that an incongruent trial is “more difficult” (Desender & Van den Bussche, 2012; Desender et al., 2013) even if the prime itself is not consciously perceived. In this case, the Gratton effect following unconscious incongruent trials could be explained in terms of adaptation to a conscious subjective experience of difficulty related to the unconscious stimulus congruency (Kinoshita, Forster, & Mozer, 2008; Kinoshita, Mozer, & Forster, 2011). This hypothesis leads to several new questions. First, even if participants are not aware of stimulus congruency, can they still have a subjective experience of difficulty, related to this unconscious congruency? Second, if so, then how is this subjective experience constructed? We need to know to which effect(s) or by-product(s) of the congruency the subjective experience is sensitive. Finally, is such a subjective experience of difficulty responsible for the Gratton effect? In the next sections, we discuss what has been described in the literature so far.

## Subjective experiences of (unconscious) congruency

Morsella et al. (2009) conducted a Stroop task where, after each trial, participants were asked to judge on an 8-points scale the strength of their urge to make an error (hereafter called urge-to-err) (Morsella et al., 2009 Experiment 1, 2 and 3). A stronger urge-to-err was reported on incongruent Stroop trials versus congruent trials. According to the authors, this result shows that participants have a subjective experience related to the response competition triggered by incongruent trials. However, participants were completely aware of the congruency of the stimulus. Therefore, participants could merely use the visual stimulus congruency to judge their urge-to-err. To dissociate whether the reported urge-to-err reflected the perceived stimulus congruency or response competition, Morsella et al. used a Flanker task (2009, Experiment 4A) with four targets and two responses. Participants had to respond to a central target letter (S, M, P or H), by pressing left for S and M, and right for P and H. On “stimulus conflict trials”, the flanking letters were different from the target, but associated with the same response as the target. (e.g., in SSMSS). In “stimulus + response conflict trials”, the flanking letters were different *and* associated with another response (e.g., in SSPSS). Participants reported a higher urge-to-err on stimulus + response conflict trials than on stimulus conflict trials. The authors argued that when processing incongruent trials, participants can consciously perceive the stimulus congruency, but would additionally have a subjective experience related to response competition. Nevertheless, in the Flanker task, again participants are fully aware of the different types of congruency. Therefore, it is again possible that participants used the perceived congruency to judge their urge-to-err.

Tackling this issue, Desender, Van Opstal, and Van den Bussche (2014) used a subliminal priming conflict task, similar to the task used by Kunde (2003) explained previously (see ***Figure 3***). After having responded to the target (i.e. first-order task), participants were additionally asked to report whether they thought that the prime and the target were pointing in the same direction or not (second-order task). Because the prime was presented close to the awareness threshold, most of the time participants could not use their awareness of the prime to judge the congruency. To give an answer, participants were instructed to use “*slowed RTs, error proneness, or a vague feeling that something was not right*” (Desender et al., 2014, p. 2). Remarkably, participants correctly classified the congruency of the trials above chance level even if unaware of the prime. Later, using the same priming task, Desender et al. (2016) showed that participants reported a stronger experience of difficulty on incongruent than on congruent trials. Additionally, and still in a similar subliminal priming conflict task, Wenke, Fleming, and Haggard (2010; see also Chambon & Haggard, 2012) showed that participants reported less experience of control on the consequences of their response on incongruent compared to congruent trials.

Looking at these results concomitantly, it seems that participants can judge incongruent trials differently from congruent trials, even if unaware of the stimulus congruency itself. Participants can report their experience of urge-to-err (Morsella et al., 2009), their experience of control (Chambon & Haggard, 2012; Wenke et al., 2010), their experience of difficulty (Desender et al., 2016), or their subjective evaluation of the congruency of a trial without being aware of the stimulus presented (Desender et al., 2014). In other words, participants would have a conscious experience of “processing fluency”, an experience that would be modulated by the congruency of the trial (e.g., Chambon & Haggard, 2012; Desender, Van Opstal, Hughes, & Van den Bussche, 2016). Processing fluency is a very general term used to refer to a subjective ease or difficulty with which information is processed or a cognitive task is performed (Oppenheimer, 2008). When reporting subjective experiences of processing fluency or task difficulty, participants are making metacognitive judgements (Chambon & Haggard, 2012). Metacognition is a broad term introduced by Flavell (1979) and is often defined as “cognition about cognition”. In the current context, when we speak about metacognition, we narrow the definition to our ability to explicitly or subjectively monitor and evaluate an ongoing cognitive process. We do not have direct objective measures of the subjective experience of another person. The most direct access we can have, is simply to ask a participant to take an introspective look at his or her own subjective experiences and to provide us with an introspective report (for a related discussion, Timmermans & Cleeremans, 2015).

Metacognitive judgements in the context of conflict tasks are an emerging topic in the literature. Researchers recently started investigating how and when such metacognitive judgements are created and whether they are used to install cognitive adaptation. A longer research tradition exists on metacognitive judgements in the field of memory and decision-making (for an overview, see Fleming & Dolan, 2012). Possibly, these findings can help us to better understand the relation between cognitive control and metacognition.

## Metacognitive judgements in decision-making and memory research

In the domain of decision-making, people are often asked to make confidence judgements on their own decisions (e.g., Fleming, Huijgen, & Dolan, 2012). For instance, participants categorise an ambiguous stimulus as belonging to one or the other category and then judge their confidence in this categorisation. It was first suggested that the confidence judgement was based on the same information as the one that leads to the categorisation of the stimulus (Galvin, Podd, Drga, & Whitmore, 2003; Vickers, 1979). Several theories on performance in perceptual decision tasks were developed based on the drift diffusion model (Ratcliff & Mckoon, 2008). This model suggests that, to create a binary categorisation of an ambiguous stimulus, evidence coming from visual processing accumulates with time. When this evidence accumulation crosses a threshold, a decision is taken. The confidence judgement would then depend on the same evidence accumulation as the one used to reach the decision (e.g., Galvin et al., 2003; Kiani & Shadlen, 2009). However, dissociations have been observed between the performance in the categorisation task and the subsequent metacognitive judgement (e.g., Del Cul, Dehaene, Reyes, Bravo, & Slachevsky, 2009; Fleming et al., 2015; Rahnev et al., 2011; Spence, Dux, & Arnold, 2016; Wilimzig et al., 2008). A very intuitive example of such a dissociation is the observation that after a decision, you end up changing your mind (e.g., Resulaj, Kiani, Wolpert, & Shadlen, 2009). To explain changes of mind, it was suggested that the accumulation of evidence could continue after the first-order decision has been taken (e.g., Pleskac & Busemeyer, 2010; Van den Berg et al., 2016). Another possibility is that the confidence judgement is based on information other than that used to make the decision (e.g., Fleming & Daw, 2017; Pasquali, Timmermans, & Cleeremans, 2010; Wokke, Cleeremans, & Ridderinkhof, 2017). Recently, it was observed that confidence judgements are more accurate when the judgement takes place *after* the response to the first-order task rather than *before* (Siedlecka, Paulewicz, & Wierzchoń, 2016). Additionally, Kiani, Corthell, and Shadlen (2014) reported that confidence judgements are also influenced by the reaction time needed to make the decision. Together, these results suggest that confidence judgements are based, not only on perceptual information coming from the stimulus, but also on information contained within the response itself (see also Fleming & Daw, 2017; Murphy, Robertson, Harty, & O’Connell, 2015).

In the domain of memory, it has been observed that people can judge, when learning an item, the explicit belief about how successful the recall will be on subsequent testing (i.e., judgement of learning) (e.g., Nelson & Dunlosky, 1991; Vesonder & Voss, 1985). They can also judge, during the recall, how confident they are in the accuracy of this recall (e.g., Juslin, 1994; Kelley & Lindsay, 1993) or whether they know the answer to a response, even if they are not able to recall it at the present moment (i.e., feeling of knowing and tip-of-tongue) (e.g., Brown & McNeill, 1966; Hart, 1965; Schwartz & Metcalfe, 1992). A first ‘direct account’ suggested that metacognitive judgements rely on a direct access to the memory traces (Hart, 1965). When asked to make a judgement of learning, learners would directly read out the strength of the memory trace that is currently formed (Cohen, Sandler, & Keglevich, 1991). This theory looks very much like the one in decision-making that suggests that confidence judgements are directly based on the same information as the first- order task. A direct account predicts a strong relation between the metacognitive judgements and the performance in the memory task itself. As with confidence judgements, dissociations between metacognitive judgements and memory performances were observed. Think for instance of the memory illusions where participants are convinced that they remember having memorized an item but never learned it (e.g., Jacoby & Whitehouse, 1989). Accordingly, an alternative hypothesis, the ‘inferential’ account, was proposed. The inferential account suggests that metacognitive judgements on memory result from inferences based on different internal and external sources of information (Kelley & Jacoby, 1990; Koriat, 1997; Schwartz, 1994). The hypothesis that several sources of information are used to create metacognitive judgements on memory, is very similar to the theories on decision-making (e.g., Siedlecka et al., 2016; Wessel et al., 2011).

The similarity in the observations made for confidence judgements in decision making and for metacognitive judgements on memory performance can bring interesting elements to the understanding of metacognitive judgements in general and to the processing of fluency in conflict tasks more specifically. First, metacognitive judgements would rely, at least partly, on post-response processes (Yeung & Summerfield, 2012, 2014). Consider for instance the case of error detection in the context of conflict tasks. Error detection refers to the ability to recognise our own errors in the absence of external feedback (Rabbitt, 1967). While confidence judgements call for the reporting of the likelihood of a correct response, error detection asks participants to report the likelihood of an error (Yeung & Summerfield, 2012, 2014). Several theories explain our ability to detect errors (for an overview, see Ullsperger et al., 2014). It has been suggested that after an error, post-response cognitive mechanisms would detect response conflict between the current incorrect response and the correct response subsequently activated (Yeung et al., 2004), a mismatch between an intended action and the actual action (Bernstein, Scheffers, & Coles, 1995; Falkenstein, Hohnsbein, Hoormann, & Blanke, 1991), or a reward prediction error (Alexander & Brown, 2011; Brown & Braver, 2005; Holroyd & Coles, 2002; Holroyd, Yeung, Coles, & Cohen, 2005; Silvetti et al., 2011). Regardless of the exact interpretation, as with confidence judgements (Yeung & Summerfield, 2012, 2014; see also Boldt & Yeung, 2015), error detection seems to rely on post-response processes that provide information on the quality of the current response. Furthermore, additional information from sensory input and proprioception could also contribute to error detection (Wessel, 2012; Wessel, Danielmeier, & Ullsperger, 2011).

The notion that metacognitive judgements would rely, at least partly, on post-response processes could also be an important element to explain, for instance, how participants are able to judge the congruency of a stimulus while the congruency itself is presented subliminally (Desender et al., 2014). Participants would have no conscious access to the congruency of the stimulus itself but instead would have conscious access to the evaluation of their motor response. Another interesting element is that regardless of the exact type, metacognitive judgements seem to be created on the basis of different sources of information (e.g., Siedlecka et al., 2016; Wessel et al., 2011). Metacognitive judgements related to processing fluency in conflict tasks could then also rely on several sources of information at the same time.

## Metacognitive judgements in the context of conflict tasks

**Figure 4:**
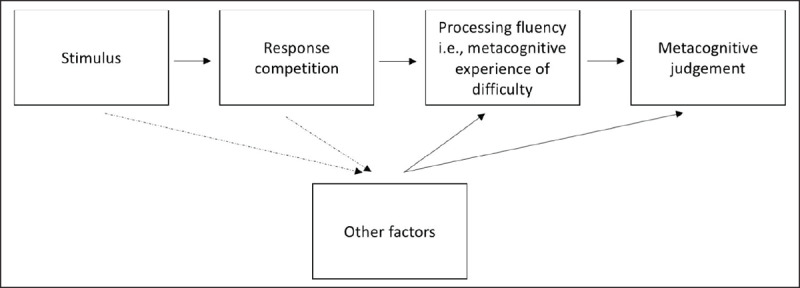
Potential sources of metacognitive judgements in conflict tasks. The metacognitive judgement would mainly rely on processing fluency (i.e., a metacognitive experience of difficulty) determined by response competition. Other factors, directly or indirectly related to the stimulus congruency, could also influence the metacognitive experience of difficulty or the metacognitive judgement of conflict.

As mentioned above, processing fluency is a general term that refers to feelings of ease or difficulty associated with mental processes. Processing fluency can be related to any cognitive process (for an overview, see Oppenheimer, 2008). Subjective experiences of processing fluency have been associated with perceptual processes (e.g., Reber, Wurtz, & Zimmermann, 2004), with linguistic processes (e.g., Alter & Oppenheimer, 2006) and/or with encoding and retrieval processes (Koriat & Ma’ayan, 2005). In the case of conflict tasks, it has been suggested that the subjective experience of processing fluency is related to the process of action-selection (Chambon & Haggard, 2012; Wenke et al., 2010). On incongruent trials, action-selection would be more difficult because of the activation of the incorrect response that triggers response competition. Participants would subjectively feel the difficulty related to this response competition. This can explain why, in a priming conflict task, participants are able to judge the congruency of a trial even if they are unaware of the prime (Desender et al., 2014), or why a congruency effect is observed on the metacognitive judgements of urge-to-err (Morsella et al., 2009), difficulty (Desender et al., 2016) or control (Chambon & Haggard, 2012; Wenke et al., 2010). In a nutshell, congruency induces response competition that would modulate the subjective experience of processing fluency, thereby determining metacognitive judgements (see ***Figure 4***).

While the idea that participants are subjectively sensitive to response competition seems a reasonable assumption, it remained a hypothesis based on indirect reasoning. It is known that incongruent trials lead to increased response competition (e.g., Hasbroucq, Possamaï, Bonnet, & Vidal, 1999). A congruency effect is also observed in several metacognitive judgements, as, for instance, in the judgement of difficulty (Desender et al., 2016). Participants associate incongruent trials with a stronger experience of difficulty compared to congruent trials. Taken together, it is inferred that the increased response competition determines the metacognitive judgements (e.g., Chambon & Haggard, 2012; Morsella et al., 2009). However, also on congruent trials, participants sometimes report an experience of difficulty (e.g., Desender et al., 2016).

To preserve the idea that response competition is the source of the metacognitive judgement of difficulty, one then needs to assume that response competition can also be induced by other “undetermined” factors (like expectations) instead of an irrelevant competing stimulus dimension (Abrahamse & Braem, 2015). There are indeed reasons to consider that response competition can also sometimes occur on congruent trials (e.g., Yeung et al., 2004; Yeung, Cohen, & Botvinick, 2011). However, response competition on congruent trials does introduce some circularity in the reasoning: “A strong experience of difficulty was reported on a congruent trial. Hence, response competition must have been present and occurred for undetermined reasons. Consequently, response competition is the source of the subjective experience and the metacognitive judgement”. While circularity does not prove the argument to be wrong, it does make it more difficulty to falsify.

Additionally, as highlighted in studies on both decision-making and memory (see above), several other factors, more or less directly related to congruency, could also influence the metacognitive judgements in conflict tasks (see ***Figure 4***). These other factors could influence the metacognitive judgements directly or because they would contribute to the subjective experience of processing fluency (Koriat, 2000; Norman et al., 2010). We already mentioned the possibility that post-response evaluations contribute to metacognitive judgements, but this possibility was not directly investigated in this context. Additionally, a difficult process is usually a slow process (for an overview, see Oppenheimer, 2008; see also Reber et al., 2004). Slower reaction times are also closely related to response competition (MacLeod, 1991). Consequently, in conflict tasks, reaction times could contribute to the subjective experience that a trial was difficult. Previous results show that reaction times do contribute but not entirely determine metacognitive judgements related to processing fluency (e.g., Chambon & Haggard, 2012; Desender et al., 2016). For instance, even when responses to congruent and incongruent trials in the same reaction time window are considered, still more difficulty is reported on incongruent compared to congruent trials (Desender et al., 2016).

Questienne, Attas, Burle and Gevers (2018) directly investigated if and how different factors (visual congruency, reaction time, response competition) contribute to metacognitve judgments. Participants were asked to perform an arrow priming task. After each trial, participants were asked to provide a subjective report of their urge-to-err (e.g. how close were you to make an error on this trial?). Electromyographic (EMG) recordings of the response hands detected the presence of partial errors. Partial errors are sub-threshold EMG activations occurring on the incorrect hand before the correct response hand is activated. Such partial errors occur on 15–20% of the trials, are more frequent on incongruent events and are regarded as a good objective measure of response competition (Burle, Roger, Allain, Vidal, & Hasbroucq, 2008). In the study of Questienne et al. (2018), both reaction time and partial errors were good predictors of urge-to-err reports, while this was not the case for the subjective reports on the visual congruency of the stimuli.

This study nicely illustrates that different factors (e.g. RT, response competition, visual congruency) contribute to metacognitive judgements. A remaining question is whether participants weight these factors differently depending on the metacognitive question. In other words, can people introspect different aspect of conflict depending on the specific question asked? In the literature, several studies are described where participants made different kinds of metacognitive judgements. For instance, in some studies, participants were asked to report their subjective experience of difficulty (Desender et al., 2016), their subjective experience of conflict (Desender et al., 2014), their subjective experience of urge-to-err (Morsella et al., 2009) or their subjective feeling of control (Chambon & Haggard, 2012; Wenke et al., 2010). In all these studies, a congruency effect was observed in the metacognitive judgements. Remarkably, regardless of the specific metacognitive judgement, these researchers interpreted this congruency effect as revealing a subjective sensitivity to response competition. This does however not need to be the case. In theory it is possible that the congruency effects in the metacognitive reports have their origin in different stages of stimulus and response processing, depending on the question. This issue was investigated by Questienne, van Dijck and Gevers (2018) who asked participants either to report their feeling of urge-to-err or their feelings of visual conflict. Demonstrating that subjective reports are valid and sensitive, they found that the subjective reports specifically followed either the response or the visual conflict. While the different factors were not measured independently in this study ((in)congruent trials always contained both visual ans reponse conflict), it is a plausible assumption that the underlying types of conflict contributed differently to the metacognitive questions asked.

In summary, it is established that participants judge incongruent trials differently than congruent trials, and while some initial steps have been taken, the origin of this/these metacognitive judgement(s) remains poorly understood. EMG recordings can serve to objectify the presence of response competition (Questienne et al., 2018). Second, several other sources could contribute to different metacognitive judgements in an implicit or explicit way, but they have not yet been identified. To give an example, research on this topic could focus on the influence of emotional valence on metacognitive reports. Finally, it is possible that different subjective judgements reported in conflict tasks reflect different experiences (Questienne, Van dijck, & Gevers, 2018) but again more work on this topic is clearly needed.

## Do conscious experiences of processing fluency trigger cognitive control?

Even though the origins and the relation between the different factors contributing to metacognitive judgements deserve more attention, it is established that participants judge incongruent trials differently from congruent trials. The question now is whether these metacognitive judgements influence the Gratton effect itself.

Consider again the study by Desender et al. (2014) where participants had to subjectively judge whether trials were incongruent or not in a subliminal priming conflict task. In this experiment, sometimes, participants judged congruent trials as incongruent and vice versa. After such trials, the Gratton effect depended on this subjective evaluation of congruency, not on the objective stimulus congruency. Using a different approach, Questienne, Van Opstal, van Dijck and Gevers (2018) reached a similar conclusion. Participants were explained that they were participating in a subliminal priming task, making it very difficult and sometimes even impossible to observe the prime. After the trial, participants were asked to indicate whether they believed the trial was a conflict trial or not. Unknownst to the particpants however, the prime was not presented on a large proportion of the trials. Regardless of the absence of the prime (and thus the absence of an objective visual conflict), a Gratton effect was observed in those trials were the participants indicated to have experienced a conflict. Together with the results of Desender et al (2014), these results suggest that there is a relation between the metacognitive judgement related to processing fluency and the Gratton effect. These findings were partly replicated by Jiang and colleagues (Jiang, Correa, Geerts & van Gaal, 2018, see also Foerster, Pfister, Reuss, & Kunde, 2017). Crucially however, they also showed that behavioral adaptation could be observed when conflict was present but not subjectively experienced. Clearly, more work on these important yet different observations is needed.

**Figure 5:**
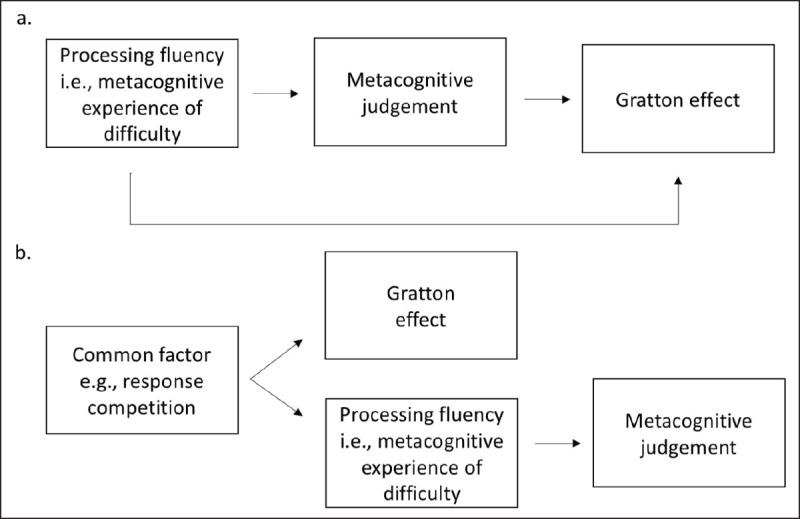
Relationship between metacognitive judgements and the Gratton effect. **(a)** causal relationship: the subjective experience and/or the related metacognitive judgement drive(s) the Gratton effect. **(b)** Correlational relationship: the subjective experience related to the metacognitive judgement and the Gratton effect have a common source (adapted from Abrahamse & Braem, 2015).

Such findings reopen the debate about the role of consciousness in the Gratton effect. Does the conscious subjective evaluation (i.e., the metacognitive judgement) play a role in conflict adaptation? As observed by Questienne, Van Opstal, Van Dijck and Gevers (2018), the mere subjective experience of processing fluency, could be important to trigger the Gratton effect (see ***Figure 5a***). An interesting case report in this respect was reported by Naccache et al. (2005). They reported the case of a patient who did not report any subjective experience of difficulty when responding to Stroop incongruent trials. Remarkably, the patient still presented a Gratton effect. While this remains a single case report, this result seems to argue against a causal role of the metacognitive judgement and/or experience in the Gratton effect. However, caution is needed for at least two reasons. First, the task used in this study was a two-choice Stroop task. As outlined before, the Gratton effect can be generated by bottom-up influence of stimulus repetition/alternation in this kind of task (for review see Duthoo et al., 2014). The observed Gratton effect could then be the effect triggered by this low-level influence, while the part of the effect triggered by top-down influence could still be impaired. Second, while this patient did not report a subjective experience of difficulty on incongruent trials, she was still able to indicate that she was responding slower on these trials. This means that she was still able to make some metacognitive judgements related to the congruency of the trials. This case report does seem to indicate that different metacognitive judgements can be dissociated. The metacognitive judgement of difficulty can be dissociated from the metacognitive judgement related to performance (i.e., reaction time). Nevertheless, it remains possible that the metacognitive judgement related to performance still plays a role in the Gratton effect.

However, the co-occurrence of metacognitive judgements and the Gratton effect on itself does not necessarily imply a causal relationship between the two (Abrahamse & Braem, 2015). It remains possible that metacognitive judgements and the Gratton effect are both triggered by a common factor without a direct link between both (see ***Figure 5b***). In this case, the relationship between subjective evaluations and the Gratton effect would only be correlational (see also Hommel, 2013).

Following this reasoning, Abrahamse and Braem (2015) argued that if the metacognitive judgement is related to the occurrence of response competition, the results obtained by Desender et al. (2014) can be explained by the conflict monitoring theory without giving any causal role to the metacognitive judgement. As illustrated in ***Figure 5b***, when a trial occurs, the trial is judged as congruent or incongruent based on the occurrence of response competition. In parallel, as suggested by the conflict monitoring theory, the level of response competition is monitored by the ACC, triggering cognitive control if response competition is detected. In most of the cases, incongruent trials are associated with response competition, while congruent ones are not. Overall then, trials would be judged correctly and a typical Gratton effect would be triggered (Botvinick et al., 2001). However, because response competition can also occur on some congruent trials because of factors other than stimulus congruency (Yeung et al., 2004, 2011), some congruent trials will be wrongly interpreted as “incongruent”. At the same time, response competition will trigger cognitive control. Superficially, the Gratton effect will look as if it follows the metacognitive judgement. But, the Gratton effect would not be caused by the metacognitive judgement. The relation is only correlational.

To understand the relation between metacognitive judgements related to congruency and cognitive control mechanisms in conflict tasks, it is essential to have a better understanding of what is really ‘felt’ during a conflict task and how this subjective experience is used to make a metacognitive judgement. More specifically, it asks us to clarify the relationship between response competition and metacognitive judgement in the context of sconflict tasks. We outlined above that participants judge incongruent trials differently from congruent trials, but that the origin of this/these metacognitive judgement(s) is poorly understood. Further research should aim to clarify the origin(s) of these metacognitive judgements related to processing fluency in conflict tasks and their relationship to cognitive control.

## Author Information

Just after finishing her PhD with Jean-Philippe van Dijck (co-promoter) and Wim Gevers (promoter) and working as a post-doctoral researcher in Marseille in the lab of Boris Burle, Laurence Questienne suddenly passed away on May 12, 2018. The current PhD critical review paper is a slightly adapted version of her PhD introduction. This work is submitted in memory of Laurence. It is therefore very well possible that some of the most recent articles on the topic are not reviewed in the current text, for which our sincere apologies.
